# Comparative Assessment of the Adaptive Behavior Among Typically Developing Children and Children With Disability at a Tertiary Care Hospital in Central India: A Cross-Sectional Study

**DOI:** 10.7759/cureus.91431

**Published:** 2025-09-01

**Authors:** Nikita Yadav, Binu Mathew, J Jeayareka, Sharda Pasari, Sheetal LNU

**Affiliations:** 1 Psychiatric Nursing, All India Institute of Medical Sciences, New Delhi, New Delhi, IND; 2 College of Nursing, All India Institute of Medical Sciences, Raipur, Raipur, IND; 3 Medicine, All India Institute of Medical Sciences, Raipur, Raipur, IND; 4 Psychiatry, All India Institute of Medical Sciences, Raipur, Raipur, IND; 5 Psychiatric Nursing, All India Institute of Medical Sciences, Raipur, Raipur, IND

**Keywords:** adaptive behaviour, autism spectrum disorder (asd), child development, communication skills, intellectual disability

## Abstract

Introduction: Globally, over 240 million children live with disabilities, facing significant barriers to early education, healthcare, and social inclusion. In India, developmental delays and neurodevelopmental conditions such as autism spectrum disorder (ASD), intellectual disability (ID), and hearing impairment (HI) affect a growing number of children. Adaptive behavior, comprising conceptual, social, and practical skills, is a critical indicator of functional development.

Objective: To assess and compare the adaptive behavior of typically developing children with that of children diagnosed with disabilities attending outpatient services in a tertiary care center.

Methods: This cross-sectional comparative study was conducted at AIIMS Raipur with a total sample of 100 children aged 2-10 years, divided equally between the disabled and typically developing groups. Participants were selected via purposive sampling. Data were collected through structured interviews using the Vineland Social Maturity Scale and analyzed using descriptive and inferential statistics, including t-tests and chi-square tests.

Results: Typically developing children scored significantly higher across all domains of adaptive behavior (p < 0.001). The widest gap was noted in self-dependence (SD), followed by self-help dressing and communication. Among the disabled subgroups, children with autism showed the lowest communication scores (p = 0.011). Demographic factors such as disability history were significantly associated with adaptive outcomes.

Conclusion: Children with disabilities, particularly those with autism spectrum disorder (ASD), exhibit markedly reduced adaptive functioning across several domains.

## Introduction

Around the globe, 240 million children live with disabilities [1]. These children are 24% less likely to receive early stimulation, 49% more likely to have never attended school, and 51% more likely to report feelings of unhappiness [1]. In India, around 2.5-3.4% of children exhibited developmental issues, with the most common being speech delay and global developmental delay [2]. The prevalence of autism spectrum disorder (ASD) in India is estimated at 1 in 500, while attention-deficit/hyperactivity disorder (ADHD) affects 11.3% of primary school children, often co-occurring with behavioral difficulties [3].

Disabilities such as intellectual disability, ASD, visual and hearing impairments, and locomotor disabilities impact a child's ability to acquire, retain, and demonstrate age-appropriate adaptive behaviors [4]. Such children frequently face barriers to accessing quality education, healthcare, and inclusive community services. Parents of children with disabilities encounter multifaceted challenges like physical exhaustion from caregiving, financial strain due to specialized services, and emotional burnout. Moreover, public spaces are often inaccessible, limiting the family's social participation [5]. The parenting demands increase significantly, requiring not just emotional strength but also knowledge of resources, therapeutic interventions, and advocacy.

Adaptive behavior refers to the collection of conceptual, social, and practical skills learned by individuals to function effectively in daily life [6]. It includes conceptual skills (e.g., communication, functional academics), social skills (e.g., interpersonal relationships, self-esteem, social responsibility), and practical skills (e.g., personal hygiene, safety, domestic activities) [7]. Adaptive behavior is age-dependent, as societal expectations and developmental milestones evolve. Typically developing children tend to acquire these behaviors in line with their age, shaped by environmental influences, parenting styles, and access to education.

There is a complex relationship between specific disabilities and adaptive behavior. For example, children with autism, especially those with average or above-average intelligence, often exhibit significant impairments in socialization and communication despite cognitive competence [8]. This study aimed at assessing the difference between the adaptive behavior of typically developing children and children with disabilities. Assessing adaptive behavior in both populations can contribute to early detection and prevention efforts, improve inclusive education, and ensure that support systems are tailored to the actual needs of children and their families.

## Materials and methods

Study approach

The quantitative research approach was considered as an appropriate research approach to accomplish the main objectives for the present study.

Study design

This is a comparative cross-sectional study. The data were analyzed in terms of the objectives of this study, using descriptive and inferential statistics. The data was organized in a master sheet and then computed using the analysis software.

Study setting 

This study was conducted among the children (2-10 years) attending the outpatient Department of Psychiatry and Pediatrics from September 2021 to December 2021, All India Institute of Medical Sciences, Raipur, Chhattisgarh, India.

Study population 

The study population included disabled and typically developing children between the age of 2 and 10 years.

Inclusion criteria

The present study included children whose parents were willing to give information, who were present and available at the time of data collection, whose parents knew either English or Hindi, whose age was between 2 and 10 years, and who were already diagnosed with the specified disability, i.e., hearing impairment, intellectual disability, or autism spectrum disorder.

Exclusion criteria

The present study excluded children whose parents were not willing to give information or who were severely ill at the time of data collection.

Sample size

The sample was calculated at 50 children per group using the following formula:

$$n = \frac{Z^2 \cdot p \cdot (1-p)}{d^2}$$

Considering a non-response rate of 10%, the sample size was rounded off to 50 children per group.

Sampling technique

**Figure 1:**
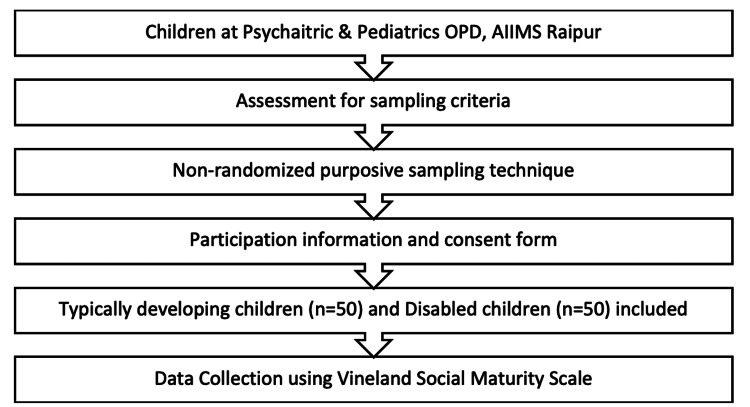
Study flow The image is created by the author.

A non-probability purposive sampling method was used to select participants based on personal judgments about which ones would be most informative (Figure 1).

Data collection technique

Formal administrative permission was obtained from the ethical committee and respective settings in Raipur, from where data were collected, and the subjects were selected according to the selection criteria of the study. Consent was taken from the parents of the child at the time of data collection. The data were taken from parents or guardians who are spending more time with the child with the structured tool, i.e., the Vineland Social Maturity Scale (VSMS), and socio-demographic variables. To obtain the social age, the raw score is converted to social age using tables and charts provided in the VSMS manual. Social age (SA) represents the age level at which the individual’s social skills are typically observed. The data were collected through standardized interviews conducted in a face-to-face interaction setting within a hospital (psychiatric OPD and pediatric OPD of AIIMS Raipur).

These settings were chosen according to feasibility and the pandemic situation. The Vineland Social Maturity Scale was originally devised by E. A. Doll in 1935. It is a useful instrument for measuring the social maturity of children and young adults. Content validity refers to the degree to which an assessment instrument is relevant to, and representative of, the targeted construct it is designed to measure. The tool was given to six experts along with a blueprint and a criteria checklist. The selection of the experts was done based on their experience, clinical expertise, and their interest in the topic selected. The experts were requested to give their opinion and suggestions regarding the appropriateness and relevance of the question. The corrections and suggestions were incorporated in the main study.

Domains of VSMS

The Indian adaptation of the Vineland Social Maturity Scale assesses social competence across eight domains: self-help general, self-help eating, self-help dressing, self-direction, occupation, communication, locomotion, and socialization.

Scoring system

Each item is scored as “Yes” (1) if the child can perform the behavior, or “No” (0) if not. Scores are added across items in each domain → raw scores. These raw scores are then converted into a social age (SA) using standard norms.

Social quotient (SQ) is then calculated as:

SQ=SA/CA×100

Where:

SA = Social Age (from scale)

CA = Chronological Age (actual age in months/years)

Ethical considerations

Ethical permission was obtained from AIIMS Raipur’s Institutional Ethical Committee, with the reference number 1746/IEC-AIIMSRPR/2021. The permission was obtained from the director, dean, and medical superintendent of AIIMS Raipur. The permission was also obtained from the head of the department of pediatrics and the psychiatric ward for data collection from the participants who met the inclusion and exclusion criteria. Participants were provided with an information sheet and informed about the study. Informed consent was taken through the participant consent form after a detailed explanation. It was assured to the participants that the confidentiality of their details would be maintained, and the data would not be used for any purpose other than research. Anonymity was assured to each participant and maintained by the researcher.

Procedure for data collection 

Data was collected after obtaining permission from the concerned authority of the IEC Committee, AIIMS, Raipur, from the MS of AIIMS Raipur, by explaining the purpose of the study. After getting permission, the investigator introduced herself to the participants and explained the study objectives to them in detail. All the participants were provided with assurance that their confidentiality to be maintained. Participants were screened for their fitness, both physically and psychologically. Informed consent and assent were taken from the subjects. The data was collected from the 100 children (50 were normal and 50 were disabled) based on the fulfillment of the inclusion criteria and their interest in participating in the study. The samples were selected by a non-probability sampling technique. The Vineland Social Maturity Scale was used to assess the adaptive behavior of the children.

Data analysis

The data was organized in an MS Excel sheet and then computed using IBM Corp. Released 2020. IBM SPSS Statistics for Windows, Version 26. Armonk, NY: IBM Corp. The data were analyzed using descriptive and inferential statistics. Continuous and categorical variables were expressed as mean ± SD and frequency and percentages, respectively. An independent t-test was used to compare the mean activity of the samples in all eight domains. A level of significance with a p-value <0.001 was considered to be highly significant.

Quality assurance

The primary author interviewed all the parents face-to-face personally to maintain the quality of data. The primary author entered all the data after removing the identifiers like name and unique patient ID. All such records were removed from the final analysis that had missing variables, and only complete records were analyzed.

## Results

The study included 100 children, 50 in each group. Both groups had a similar age distribution, with the largest numbers (n=20, 40%) in the 2-4-year age group. The disabled group had a higher proportion of males (n=31, 62%). Most children in both groups were firstborns and came from nuclear families. A majority across both groups followed Hinduism and resided in rural areas. Fathers were predominantly aged 31-40 years, while mothers in the typically developing group were mostly younger, between 21 and 30 years. Educational attainment showed that parents of disabled children were more likely to have completed graduation or postgraduate studies, whereas diploma-level education was most common among the other group. In terms of occupation, private-sector jobs were the most common across both, but government employment was higher among parents of typically developing children. Income levels were largely modest, with nearly half of both groups earning between INR 10,000 and INR 30,000 per month (Table 1).

**Table 1:** Demographic details of the typically developing and disabled children’s groups

Demographic Variables	Disabled group (n=50)		Typically developing children (n=50)	
	Frequency	Percentage (%)	Frequency	Percentage (%)
Age in years				
2-4 years	20	40%	20	40%
4-6 years	13	26%	13	26%
6-8 years	10	20%	11	22%
8-10 years	7	14%	6	12%
Gender				
Male	31	62%	25	50%
Female	19	38%	25	50%
Birth Order of the child				
First	36	72%	32	64%
Second	10	20%	12	24%
Third	3	6%	6	12%
> Three	1	2%	0	0%
Type of family				
Nuclear family	32	64%	35	70%
Joint family	17	34%	15	30%
Extended family	1	2%	0	0%
Religion				
Hinduism	43	86%	41	82%
Muslim	7	14%	9	18%
Residential area				
Urban	19	38%	15	30%
Rural	31	62%	35	70%
Age of the father				
21-30 years	9	18%	16	32%
31-40 years	35	70%	33	66%
>41 years	6	12%	1	2%
Age of the mother				
21-30 years	23	46%	37	74%
31-40 years	26	52%	11	22%
>41 years	1	2%	2	4%
Education				
Primary school	4	8%	1	2%
Middle school	8	16%	5	10%
High School	6	12%	10	20%
Diploma	7	14%	17	34%
Graduate	19	38%	15	30%
Postgraduate	6	12%	2	4%
Occupation				
Government job	11	22%	20	40%
Private job	31	62%	23	46%
Self-employed	7	14%	7	14%
Unemployed	1	2%	0	0%
Monthly Income (in INR)				
<10,000	17	34%	22	44%
10,000-30,000	25	50%	22	44%
30,001-50,000	5	10%	6	12%
>50,000	3	6%	0	0%

The comparative analysis between disabled and typically developing children reveals significant disparities across all developmental domains, each with high statistical significance (p < 0.001). Typically developing children consistently scored higher in every area measured. The most pronounced gap was observed in self-dependence (SD), where typically developing children had a mean score nearly four times that of disabled children (86.96 vs. 18.18). Similarly, large differences were seen in domains like self-help dressing (SHD) and communication (COM), indicating that children with disabilities face substantial challenges in fundamental life skills. Domains such as self-help grooming (SHG), locomotion (LOC), and socialization (SOC) also showed meaningful contrasts, though comparatively less extreme (Table 2).

**Table 2:** Comparison of mean % of activity done by disabled and typically developing children (domain-wise) SHG: Self-help general, SHE: Self-help eating, SHD: Self-help dressing, SD: Self-dressing, OCC: Occupation, COM: Communication, LOC: Locomotion, SOC: Socialization Student’s t-test was used;**p<0.001 is highly significant

Domain	Disabled children (n=50)		Typically developing children (n=50)		Mean Difference	p-value
	Mean	SD	Mean	SD		
SHG	85.50	16.90	97.57	05.61	12.07	<0.001**
SHE	69.83	21.30	96.50	06.87	26.67	<0.001**
SHD	39.97	24.80	87.66	15.25	47.69	<0.001**
SD	18.18	36.33	86.96	34.43	68.78	<0.001**
OCC	63.15	17.28	93.63	08.23	30.48	<0.001**
COM	52.98	15.28	95.17	07.70	42.19	<0.001**
LOC	75.25	17.63	98.61	05.87	23.36	<0.001**
SOC	63.90	21.32	96.21	07.59	32.31	<0.001**

In self-dependence (SD), children with autism exhibited the lowest scores, with a mean of zero, compared to higher averages in the ID and HI groups, but it was not significant. Occupational skills (OCC) showed a modest but non-significant trend, with the autism group trailing slightly. The only domain with a meaningful statistical difference was Communication (COM) (p = 0.011), where children with autism showed notably lower mean scores compared to both ID and HI groups (Table 3).

**Table 3:** Comparison of mean % of activity done by disabled groups (three groups) among all eight domains SHG: Self-help general, SHE: Self-help eating, SHD: Self-help dressing, SD: Self-dressing, OCC: Occupation, COM: Communication A one-way ANOVA test was used; *p<0.05 is significant

Domain	ID (N=21)		HI (N=14)		Autism (N=15)		p-value	Post Hoc
	Mean	SD	Mean	SD	Mean	SD		
SHG	85.67	8.969	91.41	27.76	79.87	10.04	0.187	-
SHE	70.78	18.65	76.89	20.85	61.85	23.83	0.159	-
SHD	40.86	29.34	45.18	20.35	33.87	21.67	0.469	-
SD	18.75	35.94	33.33	57.73	0	0	0.551	-
OCC	65.86	16.32	67.79	19.60	55.04	14.34	0.087	-
COM	58.15	14.62	55.44	13.72	43.44	13.90	0.011*	Autism vs. ID, HI

In every age group, children without disabilities consistently demonstrate higher mean scores, with statistically significant differences in each category. For instance, among 2-4-year-olds, typically developing children had a much higher mean score (42.75) compared to children with ID (25.30), HI (30.88), and autism (24.25). The trend continues in older age brackets, such as the 6-8 and 8-10-year groups, where autistic children showed the lowest or even no scores at all, indicating developmental delays or functional limitations in those age bands (all p-values of < 0.005) (Table 4).

**Table 4:** Comparison of adaptive behavior in terms of social age-wise A one-way ANOVA;**p<0.001 is highly significant;*p<0.05 is significant

Age	Normal		ID (N=21)		HI (N=14)		Autism (N=15)		p-value
	Mean	SD	Mean	SD	Mean	SD	Mean	SD	
2-4 years	42.75	13.04	25.30	12.75	30.88	6.78	24.25	5.25	<0.001**
4- 6 years	56.85	10.01	35.56	13.26	34.22	9.72	29.48	5.81	<0.001**
6-8 years	84.40	32.94	37.50	11.68	47.97	25.20	0	0	0.004*
8 -10 years	101.96	11.82	63.62	13.81	0	0	28.35	14.77	<0.001**

**Figure 2:**
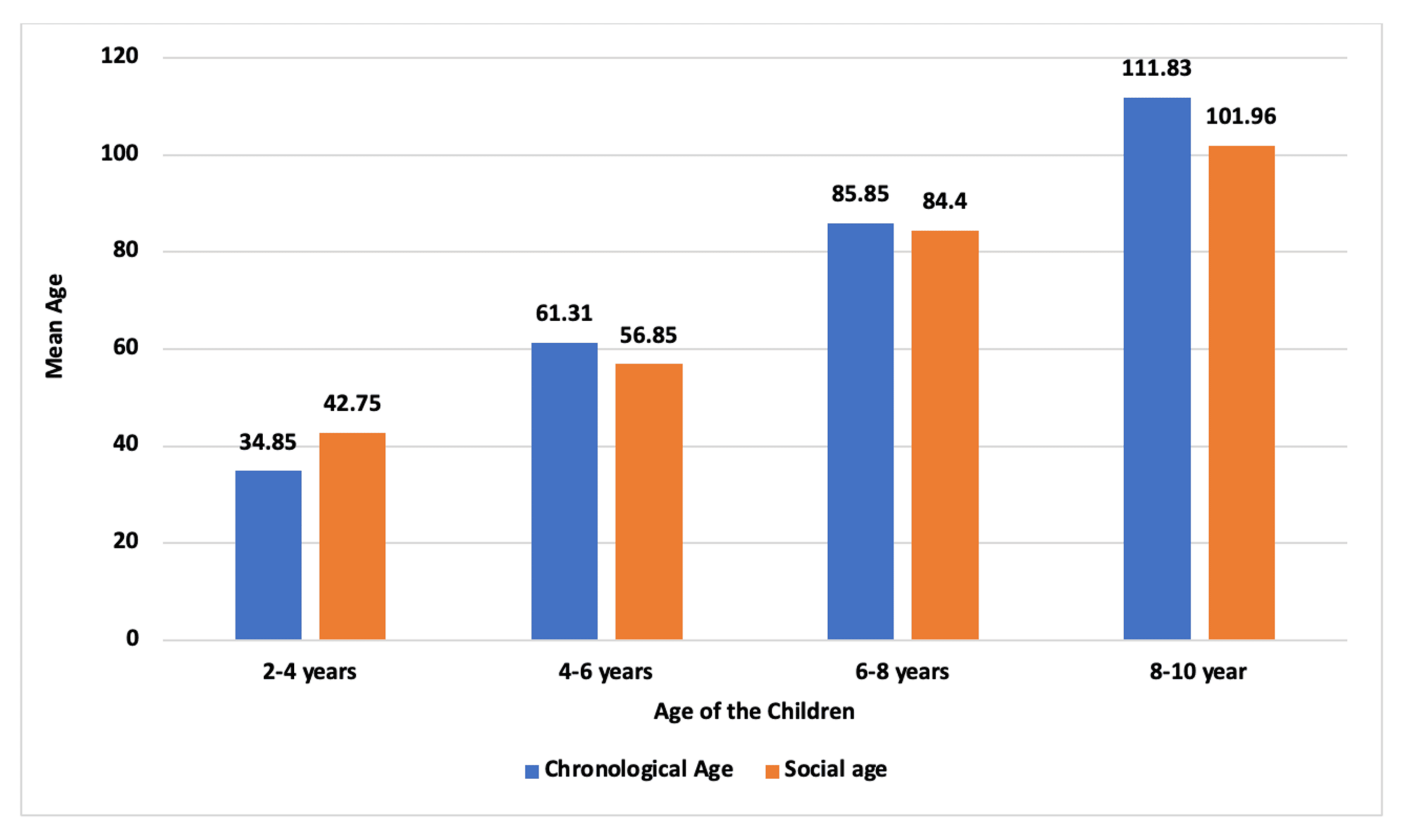
Mean of chronological and social age of typically developing children The image is created by the author.

In the youngest group of typically developing children (2-4 years), children's social age (mean: 42.75 months) exceeds their chronological age (mean: 34.85 months). However, for 4-6-year-olds, the average social age (56.85 months) lags the chronological age (61.31 months). Among 6-8-year-olds and 8-10-year-olds, social age is similar to the chronological age (Figure 2). 

Most demographic characteristics showed no statistically significant association with adaptive behavior outcomes, indicating broad similarity between the groups in terms of age distribution, gender, family type, religion, and socioeconomic status. However, disability history (χ² = 13.31, p = 0.01), where a significantly larger proportion of typically developing children had no prior disability history compared to their counterparts (Table 5).

**Table 5:** Association of adaptive behavior among typically developing children and children with disability with socio-demographic variables The Chi-square test was used; **p<0.001 is highly significant; *p<0.05 is significant

Demographic variables		Adaptive behavior				df	Chi-square test, p-value
		Disabled Children (n=50)		Typically developing children (n=50)			
		n	%	n	%		
Age	2-4 years	20	40.00%	20	40.00%	3	X^2^=0.13 p=0.99
	4-6 years	13	26.00%	13	26.00%		
	6-8 years	10	20.00%	11	22.00%		
	8-10 years	7	14.00%	6	12.00%		
Gender	Male	31	62.00%	25	50.00%	1	X^2^=1.46 p=0.23
	Females	19	38.00%	25	50.00%		
Disability History	No	29	58.00%	45	90.00%	1	X^2^=13.31 p=0.01
	Yes	21	42.00%	5	10.00%		
Birth order	First	36	72.00%	32	64.00%	1	X^2^=0.73 p=0.39
	Second	14	28.00%	18	36.00%		
Type of family	Nuclear family	32	64.00%	35	70.00%	2	X^2^=0.41 p=0.53
	Joint family	18	36.00%	15	30.00%		
Religion of the family	Hindu	43	86.00%	41	82.00%	1	X^2^=0.30 p=0.59
	Muslim	7	14.00%	9	18.00%		
Residential area	Urban	19	38.00%	15	30.00%	1	X^2^=0.71 p=0.40
	Rural	31	62.00%	35	70.00%		
Age of the father	21-25	8	16.00%	15	30.00%	2	X^2^=4.40 p=0.11
	26-30	31	62.00%	30	60.00%		
	31-35	11	22.00%	5	10.00%		
Age of the mother	21-25	19	38.00%	27	54.00%	2	X^2^=5.10 p=0.08
	26-30	25	50.00%	14	28.00%		
	31-35	6	12.00%	9	18.00%		
Education	Diploma	11	22.00%	18	36.00%	2	X^2^=2.73 p=0.26
	Graduate	27	54.00%	20	40.00%		
	Postgraduate	12	24.00%	12	24.00%		
Occupation	Government job	11	22.00%	16	32.00%	2	X^2^=1.63 p=0.44
	Private job	31	62.00%	25	50.00%		
	Self-employed	8	16.00%	9	18.00%		
Income		17	34.00%	22	44.00%	3	X^2^=1.11 p=0.57
	Rs. 10,001-30,000	25	50.00%	22	44.00%		
	Rs. 30,001-50,000	8	16.00%	6	12.00%		
	>Rs.50,000	0	0.00%	0	0.00%		

## Discussion

The present study findings reveal that there is a highly significant difference in mean activity done by disabled and normal children in all eight domains. In the disabled group, there is impairment seen in socialization, communication, locomotion, occupation, self-help dressing, self-direction, self-help eating, and self-help general domain. In the self-help dressing domain, major impairment was found in drying hands and washing the face unattended. When comparing the SHD domain between the normal and disabled groups, the main differences were seen in combing or brushing hair and bathing self-unattended in the disabled group. Typically developing children showed slight impairment in self-help dressing and self-direction, but no significant impairment was seen in other domains. The present findings were consistent with the study by Sadrossadat et al., which showed that the following domains were significantly lower in mentally retarded than in normal individuals: independent functioning, economic activity, language development, number and time, prevocational/vocational activity, self-direction, responsibility, socialization, disturbing interpersonal behavior, domestic activity, social engagement, conformity, and trustworthiness [9].

The chronological and social age of typically developing children was not the same between the 2-8 and 8-year age groups, which means that the typically developing children were lacking somewhere in their development; hence, equal attention should be given to typically developing children. Between the 2-4-year-old age group, the social age was more than the chronological age, which signifies that proper care and attention can improve adaptive behavior. Conversely, in the age groups of 4-6 years and 8-10 years, the social age was less than the chronological age, which may be due to a lack of care and training by the parents, or there are certain environmental factors that also influence adaptive behavior. The above findings were consistent with the study by Zhou et al., which found that preschool fostering type, parents' relation, social mood of living area, and mother's age and health state had a remarkable influence on the social adaptive behavior of children [10]. Hence, a good fostering type, parents' harmonious relationship, healthy children's social environment, and good health of the mother would help improve children's social adaptability [11].

Children with autism have the least adaptive behavior in terms of social age. In the ID group, there was little development between 4-6 and 6-8 years, which showed a lack of training, supervision, and knowledge among parents of how to deal with them. Among hearing-impaired children, social age varied greatly across age groups, possibly due to increased screening and use of assistive technologies, which may limit adaptive behavior development. So, the focus of care should not only be on hearing development but also on adaptive behavior, like children must be educated on various communication techniques and how to demand attention and follow simple instructions [12].

In the present study, there is a significant difference in the communication domain among all three disability groups. The autism group showed a significant difference in the communication domain when compared with ID and HI. The present findings were consistent with Mouga et al.’s study, which found that impairment in adaptive behavior within the domain of socialization skills remains a distinctive factor of ASD [13]. Another study by Ventola et al. also showed that the ASD group demonstrated significantly weaker socialization skills, including deficits in basic social behaviors [14].

In the present study, there is no statistically significant association found between demographic variables and adaptive behavior in terms of social age. The present findings were consistent with the study conducted by Lakhan et al., which shows that overall, India has a prevalence of 10.5/1000 in ID. The urban population has a slightly higher rate (11/1000) than the rural population (10.08/1000; p = 0.044). Age was found to be highly correlated with the prevalence of ID in rural children (ϱ=0.981, p = 0.019) as well as in children (ϱ = −0.954, p = 0.000) and adults (ϱ= −0.957, p = 0.000) in the urban population [15].

Recommendations

A similar study can be conducted on a large sample to generalize the finding and can be used by other researchers to assess the adaptive behavior of other disabilities also. Along with adaptive behavior, other developmental patterns, like physical, mental, and social, can also be assessed and compared. Further, it can be used to assess the effect of preventive measures to improve the adaptive behavior among the disabled children. It can also be used to assess the effectiveness of the Vineland Social Maturity Scale in order to compare the adaptive behavior among typically developing and disabled children.

Limitations

The study limitations include a single-center study with a limited sample size; hence, the findings cannot be generalized to the population. The study relies on the self-reported data; hence, it is prone to recall bias of the participants.

Implications

The findings of the study have the following implications in the areas of nursing services, nursing education, nursing administration, and nursing research:

Nursing Practice

The content of the study will help nursing professionals to focus on adaptive behavior in hospitals or in any setting. Nurses can plan various strategies to improve adaptive behavior among disabled children and typically developing children. The professional nurse can use this intervention to help disabled children adapt to the environmental situations. Nurses should plan for appropriate rehabilitation programs also.

Nursing Education

As adaptive behavior is something new to be learned, it is very important for nursing students and nurses to know about adaptive behavior. As everyone is surrounded by children, it is very important to educate nursing students about the normal development of children and how to observe the changes in their normal pattern of development. The nurse educator can also use research findings to provide evidence-based knowledge.

Nursing Administration

The nursing administrator can organize conferences, workshops, and in-service education programs regarding the various aspects of adaptive behavior among various nursing personnel. The administrators can encourage the nurses to actively participate in the rehabilitation programs or programs that will improve the adaptive behavior of both normal and disabled children. A nurse administrator can motivate their nurses to express their feelings and take help if they need it, as well as inhibit such activities in the ward.

Nursing Research

The findings of the study help to expand the scientific and professional knowledge on which further research can be conducted on adaptive behavior. This study motivates other researcher to conduct further studies to evaluate the adaptive behavior among other groups also.

## Conclusions

Adaptive behavior is significantly impaired in the disabled children group when compared with the typically developing children. In every domain, disabled children are lacking and need immediate attention and intervention. But as it is evident that typically developing children were also lacking somewhere, hence preventive measures should be taken not only for disabled children but also for the typically developing children.
